# Autoimmunity and the microbiome

**DOI:** 10.1111/imr.13363

**Published:** 2024-07-09

**Authors:** Laura M. Cox, Vijay K. Kuchroo

**Affiliations:** 1 Ann Romney Center for Neurologic Diseases, Brigham & Women’s Hospital, Harvard Medical School, Boston, Massachusetts, USA; 2 Gene Lay Institute of Immunology an Inflammation, Boston, Massachusetts, USA

**Keywords:** autoimmunity, microbiome, multiple sclerosis, rheumatoid arthritis

The gut microbiome is a diverse collection of bacteria, fungi, and viruses that have coevolved with the immune system. The microbiome plays a central role in shaping immunologic development as well as regulating other physiologic processes, including metabolic and neurologic functions. Several of the key mechanisms relate to (a) the activation of innate immune system and induction of specific immune cell subsets by pathogen-associated molecular patterns (PAMPS); (b) microbial adherence to the intestinal epithelia surface; (c) the secretion of immunomodulatory metabolites; and (d) biomimicry. While these interactions may be crucial for normal immunologic development, overactivation of these same microbe-immune signaling pathways may lead to the induction of tissue inflammation and autoimmunity.^1^ This special issue will cover the mechanisms by which the gut microbiome influences autoimmune diseases, including type 1 diabetes (T1D),^2,3^ systemic lupus erythematosus (SLE),^4,5^ rheumatoid arthritis (RA),^6,7^ and multiple sclerosis (MS).^8–10^ Also covered are considerations for host factors such as genetics, aging, and sex, as well as translation for prevention and treatment of autoimmune disease (Figure 1).

*Th17 cells* are highly responsive to the gut microbiota,^11^ play a central role in autoimmunity, and also play important roles in tissue repair, and protection against infection.^1^ Major questions in the field relate to (a) what factors determine a pathogenic versus homeostatic/protective Th17 cells and (b) what role the gut microbiota play in shaping these responses. This topic is reviewed by Schnell in this special issue.^1^ In groundbreaking work using single cell sequencing, Schnell and colleagues identified a novel stem-like and selfrenewing Th17 population denoted by TCF1^+^ transcription factor and SLAMF6^+^ receptor expression.^12^ The stem-like SLAMF6^+^ Th17 cells largely reside in the intestinal mucosa, and migrate to the intestinal mucosal following adoptive transfer. Further, they are depleted by oral antibiotics, suggesting that the gut microbiota plays an essential role in maintaining them. In models of autoimmune diseases, stem-like Th17 cells can differentiate into pathogenic CXCR6^+^ Th17 cells that traffic to the extraintestinal sites where they induce tissue inflammation (e.g., the CNS in EAE). Other studies confirm the finding of the presence of stem-like SLAMF6^+^ Th17 cells in the gut and demonstrate that they can also differentiate into IL-10 producing Th17 cells, which have anti-inflammatory functions. This first article in our special issue on the Autoimmunity and the Microbiome sets the stage to understand specific signaling mechanisms at the mucosal interface that may have broad implications in tissue homeostasis and tissue inflammation.

*Type 1 diabetes* is mediated by autoreactive effector T cells that induce destruction of β cells in the pancreas, diminishing insulin production.^2,3^ In this issue, Yau and Danska discuss immunologic mechanisms by which the early-life microbiota modifies disease risk. Fuhri, Nieuwdorp and colleagues discuss the potential of fecal microbiota transplants for treating T1D.^3^ While several genetic variations have been linked with T1D risk, including specific HLA haplotypes, the disease penetrance is variable.^2^ Studies from the Danska laboratory have shown that sex-specific microbiota interactions can shape T1D incidence in animal models.^13^ Furthermore, there are distinct geographic variations in disease prevalence, including lower rates in China, Venezuela, and Russia and higher rates in Sardinia and Finland. Because the microbiota can vary by region, researchers have hypothesized that this may be explained in part by the differences in gut microbiome.

Emerging data suggest that the first 6 months of life in humans and the first 4 weeks in mice during the nursing period represents a critical window of both immunologic^2^ and metabolic^14^ programming, in which early life microbes can shape long-term responses. In a longitudinal study of infants with increased T1D risk due to HLA haplotype, there was a higher prevalence of seroconversion to anti-insulin antibody production in children in Finland versus Russia.^15^ Seroconversion was linked to a reduction in *Bifidobacterium*, the major human-milk oligosaccharide metabolizer, a reduction in *Escherichia*, and an increase in *Bacteroides*. While both *Bacteroides* and *Escherichia* are Gram-negative LPS containing bacteria, *Bacteroides* LPS is much less immunostimulatory than *Escherichia*, suggesting that early life immunostimulation and tolerance induction may be an important mechanism for prevention of autoimmunity. In addition to the critical interactions that happen during the weaning period, the complex interplay between the microbiota, T cells, B cells, leading to auto-antibody production is expertly discussed by Yau and Danska in this issue.^2^

Studies in T1D animal models demonstrate that transferring the fecal gut microbiota can modulate disease incidence and severity, raising the question of whether similar approaches could be therapeutic for subjects with T1D or are at risk of developing disease.^3^ While this is still a relatively new approach, it is most commonly used to treat recurrent *Clostridiodes difficile* diarrhea, with greater than 90% efficacy in cases that fail initial treatment with antibiotics.^16^ While there are still limited studies in T1D, Nieuwdrop and colleagues have conducted the largest pilot FMT clinical trial to date, consisting of 10 individuals who receive healthy control allogeneic FMT versus 10 individuals who receive autologous FMT. Surprisingly, they observed better signs of insulin control in the autologous FMT group. This was accompanied to alterations in microbiota metabolites and proportions of CD4^+^ CXCR3^+^ and CD8^+^ CXCR3^+^ cells. Along with three potentially promising case studies, the authors suggest that larger FMT trials with biologically inactive placebos are the next critical step to establishing whether FMTs could be effective therapy for T1D. While FMT had early and unequivocable successes for treating *C. difficile* diarrhea,^16^ the use of FMT for chronic and immune-mediated diseases has been more challenging. This review discusses important immunologic mechanisms, donor selection, and treatment timing that may help address this unmet need.

*Systemic lupus erythematosus* is a complex autoimmune disease mediated in part by antinuclear antibodies (ANA) that cause damage in multiple tissues.^5^ The gut microbiota plays an important role in the pathogenesis of lupus by shaping immunometabolism, as reviewed by Garcia et al.^5^; these interactions show clear sex-bias, as illustrated by Lee, Chervonsky, and colleagues in this special issue.^4^ There is a dynamic interplay between the microbiota, metabolism, and the immune system.^5^ In homeostasis, lower metabolic needs from quiescent immune cells are maintained by catabolic utilization of glucose, glutamine, and free fatty acids, whereas the rapid expansion and increase in function from effector immune cells in autoimmune diseases leads to a switch toward anabolic metabolism. These metabolic changes and subsequent changes in tissue oxygenation may impact the gut microbiota and can in turn affect autoimmunity. The key mediators, including hypoxia, glucose, tryptophan derivatives, and metal ions that regulate the bidirectional interaction between the gut microbiota and SLE are discussed in detail in this special issue, as is the potential for harnessing the microbiome and FMT for the treatment of SLE.^5^

Studies using germ-free animal provide strong evidence that the gut microbiota regulates autoimmunity in multiple animal models, with divergent effects in different models of disease (see Lee et al,^4^ tab. 1 from this issue).^4^ On one hand, the absence of the gut microbiota ameliorates disease in the experimental autoimmune encephalomyelitis (EAE) model of MS, the K/BxN model of autoimmune arthritis, and in the B6SKG model of SLE,^4^ all linked to the reduction of Th17 induction by the microbiota. On the other hand, depleting the gut microbiota worsens disease in the Aire^−/−^ mice which model of autoimmune polyendocrinopathy-candidiasis-ectodermal dystrophy (APECED), in the FoxP3^+^ T cell ablation model of immune dysregulation-polyendocrinopathy-enteropathy-X-linked (IPEX) syndrome, and in the MRL-FAS^lpr^ and B6.NZM animal models of SLE. Furthermore, the presence of the gut microbiota may determine disease in a sex-specific manner. Females show a higher prevalence of autoimmune disease. In the B6.NZM SLE animal model, females show higher levels of splenomegaly, complement and IgG deposition in the kidney, and presence of ANA. Surprisingly, depleting the microbiota led to higher splenomegaly in both male and female mice, and raised the ANA levels in GF-males.^4^ This suggests that the male microbiota may play a protective role, preventing autoimmunity. Such an effect has also been observed in animal models of T1D.^13^ The major mechanisms related to sex bias include differential immunoregulation, X-linked immune genes, and the bidirectional regulation of the microbiota and androgens that ultimately impact immunity.

*Rheumatoid Arthritis* is mediated by autoreactive T and B cell responses against proteins collagen, vimentin, and fibrinogen, that lead to progressive joint damage.^6,7^ Similar to other autoimmune diseases, the etiology of RA is also multifactorial and can be modulated by both host and environmental factors. In this special issue, Lamba and Taneja discuss the intersection between genetic factors, diet, sex, and the microbiota in determining autoreactivity in RA,^6^ and Nayak and Orellana discuss how the human gut microbiome impacts disease-modifying therapies for RA and other autoimmune diseases.^7^

There is strong evidence that specific HLA haplotypes increase RA risk, which can be mediated in part by the gut microbiota, and is reviewed extensively in work from Lamba and Taneja in this issue.^6^ While levels of many gut microbes are altered in RA, mounting evidence suggests that *Eggerthella lenta* may specifically contribute to RA based on its association with higher levels of autoantibodies, rheumatoid factor, and anticitrullinated antibodies. Furthermore, *E. lenta* and another gut commensal *Collinsella aerofaciens* can worsen disease severity in the DQB1*302 (DQ8) humanized mouse model of RA. Beyond genetics, the review from Taneja et al. raises several other factors including smoking, diet, metabolites, aging, and sex-specific interactions that may influence the role of the microbiota in RA.^6^

A critical factor in treatment success in autoimmunity is effective disease-modifying therapy (DMT). The gut microbiota can affect therapeutic efficacy through several mechanisms, which are reviewed in detail by Nayak and Orellana in this special issue.^7^ The microbiota can affect efficacy of DMT via enzymatic transformation to an activated or inactivated form of the drug, by modulating circulating levels via the enterohepatic circulation, by affecting responses to immunomodulatory therapies, and by positive or negative selective pressure of the DMT on the microbiome. Approaches to identify specific mechanisms that are covered in this review include germ-free/gnotobiotic animal models, both sequencing and cultivating the microbiome, and analytical chemistry to define specific microbe–drug reactions. Specific to RA, there is a two-way interaction between methotrexate (MTX) and the microbiome, wherein the microbiome can affect MTX efficacy and side effects, and MTX can select for beneficial microbes. This framework and example provided with a focus on MTX and RA illustrates central aspects of how the gut microbiota shapes clinical outcomes in autoimmunity by affecting responses to therapy.^7^

*Multiple sclerosis* is driven by autoreactive T and B cells that attack the myelin sheath on neurons and leads to motor and cognitive deficits. While there is strong evidence that the gut microbiota can contribute to MS, these can be mediated by host factors, including genetics, diet, metabolites, and age, as mechanistically discussed in three reviews in this special issue.^8–10^ While approximately 20%–30% of MS risk is determined by genetics, the remaining 70%–80% of disease risk may be determined by environmental factors.^8^ Montgomery, Peipert, and Krementsov discuss the complex interaction between the diet, genetics, the gut microbiota, and metabolites that affect autoimmunity.^8,9^ The microbial transformation tryptophan derivatives induced Th17 production and worsened EAE, which was dependent on the genetic background of the mouse model.^17^ In addition, this review further discusses other microbial key mediators including short-chain fatty acids, amino acid derivatives, and other phenolic metabolites (see figs 1 and 2 for detailed pathways), and specific dietary interventions, microbes, and immunologic pathways that may shape autoimmune disease in the context of MS and EAE.

Because of the several confounders in MS and EAE research, the review from Peters, Gerdes, and Wekerle review the strengths of investigating mechanisms in tightly controlled twin-cohorts that are discordant for MS.^9^ Major triggers of MS that may account for discordance in disease include Epstein–Barr virus infection, an altered microbiota, altered gastrointestinal function and bowel dysfunction, diet, and obesity. In a pioneering study, this group demonstrated that there were microbiota differences in twins with multiple sclerosis versus their unaffected twin, and that transferring the MS microbiota to germ-free relapsing–remitting EAE mice worsened disease incidence while transfer of the healthy twin microbiota did not.^18^ This established not only that the gut microbiota directly contributes to autoimmunity in the context of an MS animal model, but also that the gut microbiota can regulate disease penetrance in a genetically susceptible population. The review from Wekerle and colleagues provides an in-depth look at modeling different microbiota cohorts to uncover “microbial offenders,” which could be broadly translated in the field, as well as review mechanistic pathways by which the gut microbiota contributes to disease pathogenesis across animal models of MS (see tab. 1).^9^

One of the most pressing concerns in multiple sclerosis is the development of the progressive form of the disease, and aging increases risk of this occurring. The gut microbiota changes during aging may contribute to age-related immunologic and neurologic diseases. This novel application of aging and microbiota interactions in multiple sclerosis is reviewed in detail by Fettig, Pu, Osborne and Gommerman in this special issue.^10^ Importantly, clinical outcomes show worse severity with age as well as later onset, and multiple EAE animal models show altered severity and signs of disease progression in young versus aged animals (see tab. 1 and fig. 2 of Fettig et al). Aged-adoptive transfer models of MOG33-55 or PLP-primed Th17 cells demonstrate important pathologic features of progressive MS, including cortical gray matter lesions, cortical atrophy, and microgliosis. In new work from their group, they found that transferring old versus young human microbiota from household study pairs worsened disease in the PLP-Th17 adoptive transfer model, suggesting that both biologic host and microbiota aging may contribute to disease.^19^ The review by Gommerman and colleagues further discusses specific microbial metabolites, immune mechanisms, and pathways toward FMT therapy for the aging microbiota in MS, and compares and contrasts these findings to classic age-related neurologic diseases such as Alzheimer’s disease, Parkinson’s disease, and stroke.

*As a collective issue on Autoimmunity and the Microbiome*, there is clear evidence that the gut microbiota contributes to autoimmune disease. These articles highlight the latest research that dissecting immunologic mechanisms in animal models, defining approaches for human observational studies, defining the combinatorial interactions between genetic and other host/lifestyle factors, and paving a way toward harnessing the gut microbiome in the treatment of autoimmune disease.

## Figures and Tables

**FIGURE 1:**
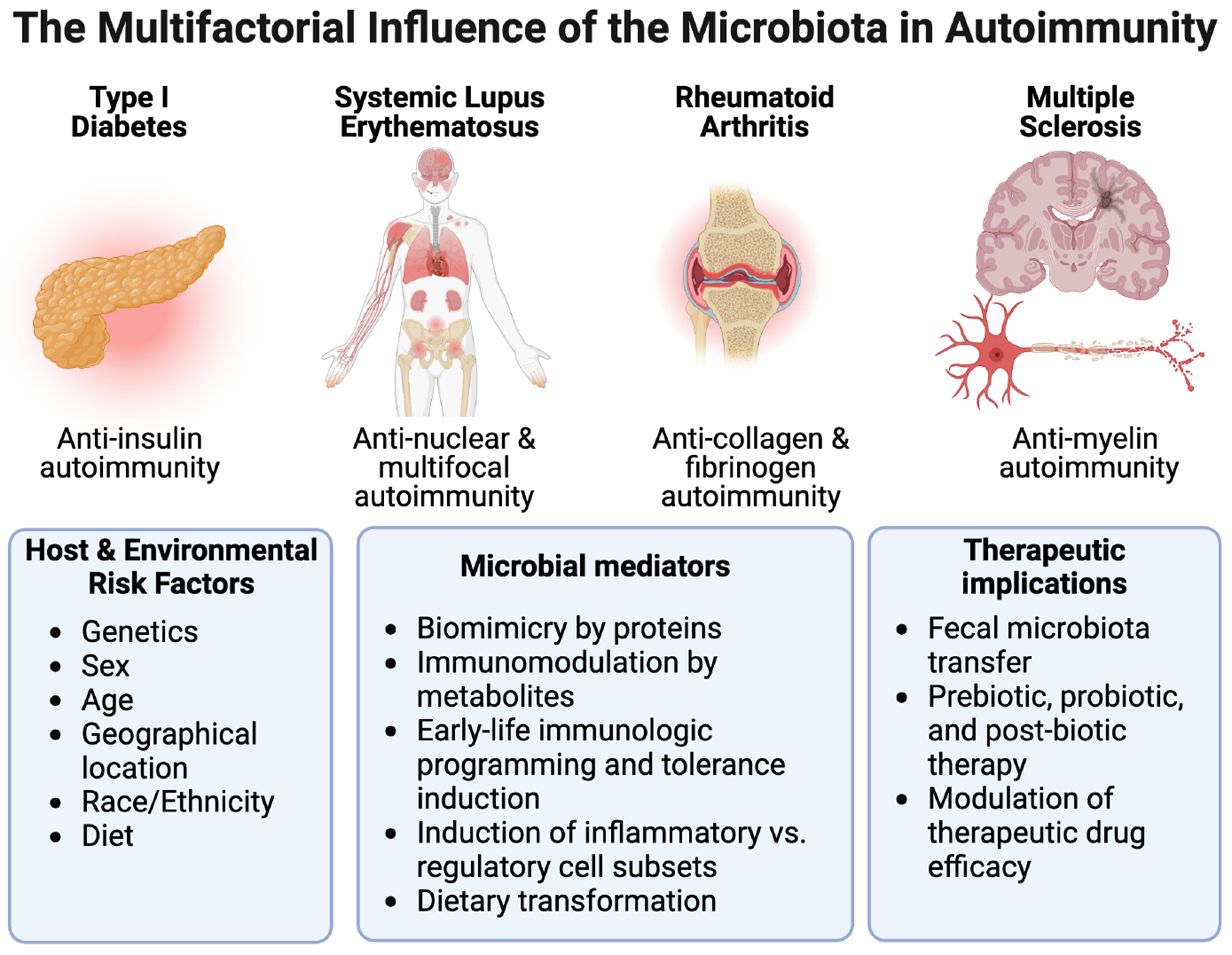
The Multifactorial influence of the microbiota in autoimmunity. While autoimmune diseases present with differential pathology in target organs, similar factors including host genetics, sex, age, geographical location, race, ethnicity, and diet can all shape risk and modulate the gut microbiota. In addition, the gut microbiota can further modify the risk of autoimmunity by triggering the disease via biomimicry, modulating inflammatory versus regulatory subsets with the production of microbial metabolites and the transformation of dietary compounds. These interactions may play critical roles in both early life immunologic programming and in aging-related exacerbations of autoimmune diseases. The gut microbiota has an important role in the clinical management of autoimmunity, both in terms potential microbiome-based therapies including FMT, prebiotics, probiotics, and post-biotics and in terms of modulating the efficacy of disease-modifying therapies.

## Data Availability

There is no original data with this study.
